# The severity of COVID-19 upon hospital admission is associated with plasma omega-3 fatty acids

**DOI:** 10.1038/s41598-024-60815-y

**Published:** 2024-05-03

**Authors:** Ligia P. Fernandes, Igor H. Murai, Alan L. Fernandes, Lucas P. Sales, Marcelo M. Rogero, Bruno Gualano, Lúcia P. Barroso, Ginger L. Milne, Rosa M. R. Pereira, Inar A. Castro

**Affiliations:** 1 Department of Food and Experimental Nutrition, Faculty of Pharmaceutical Sciences, LADAF, University of São Paulo, Av. Lineu Prestes, 580, B14, São Paulo, SP 05508-900 Brazil; 2 Bone Metabolism Laboratory, Rheumatology Division, Faculty of Medicine, University of São Paulo, São Paulo, SP Brazil; 3 Nutritional Genomics and Inflammation Laboratory, Department of Nutrition, School of Public Health, University of São Paulo, São Paulo, 01246-904 Brazil; 4 Applied Physiology & Nutrition Research Group, Faculty of Medicine, University of São Paulo, São Paulo, SP Brazil; 5 Statistics Department, Institute of Mathematics and Statistics, University of São Paulo, São Paulo, Brazil; 6 Eicosanoid Core Laboratory, Division of Clinical Pharmacology, Vanderbilt University School of Medicine, Nashville, TN USA; 7 Food Research Center (FoRC), CEPID-FAPESP, Research Innovation and Dissemination Centers São Paulo Research Foundation, São Paulo, 05468-140 Brazil

**Keywords:** COVID, Oxylpins, Fatty acids, Cytokine, Eicosapentaenoic, Diseases, Medical research, Risk factors

## Abstract

Fatty acids are precursors of inflammatory oxylipins. In the context of COVID-19, an excessive production of pro-inflammatory cytokines is associated with disease severity. The objective was to investigate whether the baseline omega 3/omega 6 fatty acids ratio and the oxylipins were associated with inflammation and oxidative stress in unvaccinated patients with COVID-19, classified according to the severity of the disease during hospitalization. This Prospective population-based cohort study included 180 hospitalized patients with COVID-19. The patients were classified into five groups according to the severity of their disease. Group 1 was the least severe and Group 5 was the most severe. Three specific types of fatty acids—eicosapentaenoic acid (EPA), docosahexaenoic acid (DHA), and arachidonic acid (AA)—as well as their enzymatic and non-enzymatic oxylipins were determined using chromatography coupled mass spectrometry. There was no difference in the ratio of omega-3 to omega-6 fatty acids between the groups (*p* = 0.276). However, the EPA/AA ratio was lower in Group 4 compared to Group 1 (*p* = 0.015). This finding was associated with an increase in both C-Reactive Protein (*p* < 0.001) and Interleukin-6 (*p* = 0.002). Furthermore, the concentration of F_2_-Isoprostanes was higher in Group 4 than in Group 1 (*p* = 0.009), while no significant changes were observed for other oxylipins among groups. Multivariate analysis did not present any standard of biomarkers, suggesting the high complexity of factors involved in the disease severity. Our hypothesis was confirmed in terms of EPA/AA ratio. A higher EPA/AA ratio upon hospital admission was found to be associated with lower concentration of C-Reactive Protein and Interleukin-6, leading to a better prognosis of hospitalized SARS-CoV-2 patients. Importantly, this beneficial outcome was achieved without any form of supplementation. The trial also provides important information that can be further applied to reduce the severity of infections associated with an uncontrolled synthesis of pro-inflammatory cytokines.

**Trial registration**: https://clinicaltrials.gov/study/NCT04449718—01/06/2020. ClinicalTrials.gov Identifier: NCT04449718.

## Introduction

Since the onset of the SARS-CoV-2 outbreak in 2020, it has been observed that while some patients only exhibited mild clinical symptoms or were even asymptomatic, others required treatment in intensive care units (ICUs)^1–5^. Furthermore, the prognosis of COVID-19 patients has been associated with a multitude of factors, with a high concentration of pro-inflammatory cytokines being one of the main features in the adverse response, resulting in a poorer outcome and/or increased mortality^5,6^.

Viral ssRNA(+) in the cytoplasm induces stress in the endoplasmic reticulum, leading to the release of fatty acids that esterify the phospholipids in the cell membrane. These fatty acids can be substrates for oxidative reactions, giving rise to various oxylipins^7^. The type of oxylipin formed, whether pro- or anti-inflammatory, depends on the fatty acid precursor and the oxidative pathway involved. Generally, oxylipins derived from enzymatic and non-enzymatic oxidation of omega-6 fatty acids, such as arachidonic acid (AA), have been found to be more inflammatory compared to those derived from omega-3 fatty acids (n-3 FA) like eicosapentaenoic acid (EPA) and docosahexaenoic acid (DHA)^6,8,9^. On the other hand, EPA and DHA are more unsaturated than omega-6 fatty acids (n-6 FA), making them more susceptible to oxidation and potentially giving rise to other cytotoxic oxylipins^10^.

Therefore, this study aimed to investigate whether the baseline ratio of omega-3/omega-6 fatty acids is associated with the inflammation and oxidative stress in non-immunized patients with COVID-19, classified according to the severity of the disease during hospitalization.

## Methods

### Study design and cohort

In this prospective population-based cohort study, randomized patients diagnosed with COVID-19 by polymerase chain reaction (PCR) test or by serology assay (ELISA) to detect IgG against SARS-CoV-2 at hospital admission were recruited from the Clinical Hospital of the School of Medicine of the University of São Paulo and the Ibirapuera Field Hospital, São Paulo, Brazil. Patients were enrolled in this multicenter study from June 5, 2020, to September 17, 2020. This study was included as an additional part of another research, thus following the same inclusion and exclusion criteria previously reported^11^ (Supplementary Fig. 1). One patient was excluded from the initial analysis because it was not possible to find the information about his discharge. All research was performed in accordance with relevant guidelines/regulations and the Declaration of Helsinki). The patients provided written informed consent before participation according to the Ethics Committee of the Clinical Hospital of the School of Medicine of the University of São Paulo and the Ethics Committee of the Ibirapuera Field Hospital (CAAE 38237320.3.0000.0068). Approved on February 12, 2021. Blood samples were collected in a fasting condition at the admission to the hospital using venipuncture into EDTA Vacutainer® tubes. Plasma samples were kept at − 80 °C until the final analysis.

### Patient stratification according to disease severity during hospitalization

This study was carried out during the first wave of COVID-19 (from June 5, 2020, to September 17, 2020). During this period, the main criteria for patients admission were: diagnosis of COVID-19 presenting respiratory rate greater than 24/min, saturation less than 93% while breathing room air, or risk factors for complications (e.g., heart disease, diabetes, systemic arterial hypertension, neoplasms, immunosuppression, pulmonary tuberculosis, obesity) followed by COVID-19 confirmation; while for patients discharge were no need for supplemental oxygen in the past 48 h, no fever in the past 72 h, and oxygen saturation greater than 93% without supplemental oxygen and without respiratory distress^11^. These criteria were similar to other hospital’s emergency department admissions during COVID-19 outbreak^12,13^. The criteria adopted to stratify patients according to disease severity were: length of hospital stay in days from the date of randomization until hospital discharge or death, need for supplemental oxygen, non-invasive mechanical ventilation, need for orotracheal intubation (OTI) in intensive care units (ICUs), and death. Based on these criteria, patients were stratified into five groups. Group 1 included patients hospitalized for less than the median, calculated as 5 days, without supplemental oxygen. Group 2 comprised those discharged until 5 days but received supplemental oxygen and/or non-invasive mechanical ventilation. Group 3 comprised patients who remained hospitalized for over a median (5 days) and needed supplemental oxygen and/or non-invasive mechanical ventilation. Finally, Group 4 included patients who were treated in intensive care units (ICUs) and received orotracheal intubation (OTI), while Group 5 was composed of patients who died during hospitalization. Thus, the severity of the disease increased from Group 1 to Group 5. The criteria deviated from the NIH-COVID-19 Treatment Guidelines^14^, since all patients under study were hospitalized. In actuality, the classification utilized in our research was suggested by physicians as the most accurate representation of the patients’ conditions.

### Determination of oxylipins

Plasma (100 µL) was placed in a microcentrifuge tube containing 500 µL 25% methanol in water and internal standard mix (1 ng each deuterated oxylipin). The sample was vortexed and spun to pellet protein. The supernatant was then extracted on an Oasis MAX uElution plate (Waters Corp., Milford, MA) as follows. Sample wells were first washed in methanol (200 µL) followed by 25% methanol in water (200 µL). The sample was then loaded into the well and washed with 600 µL 25% methanol. Eicosanoids were eluted from the plate with 30 µL 2-propanol/acetonitrile (50/50, v/v) containing 5% formic acid into a 96-well elution plate containing 30 µL water in each well. Samples were analyzed on a Waters Xevo TQ-XS triple quadrupole mass spectrometer connected to a Waters Acquity I-Class UPLC (Waters Corp., Milford, MA USA). Separation of analytes was obtained using an Acquity PFP column (2.1 × 100 mm) with mobile phase A being 0.01% formic acid in water and mobile phase B acetonitrile. Eicosanoids were separated using a gradient elution beginning with 30% B going to 95% B over 8 min at a flow rate of 0.250 mL/min. One analysis was carried out/patient and the concentration was expressed as ng/mL plasma.

### Determination of the fatty acids profile

Plasma (50 µL) were transferred to test tubes containing 0.1 mg of tricosanoic acid methyl ester as internal standard (IS) (C23:0; Fluka 91478), 20 μL of a 0.5% butylated hydroxytoluene (BHT) solution and 1 mL of a methanolic NaOH solution (0.5 M). Then, samples were placed in a boiling water bath for 5 min, followed by addition of 2 mL of boron trifluoride diethyl etherate (BF_3_) and boiling for 5 min. After cooling, 1 mL of isooctane was added and the mixture was vigorously homogenized. Then, 5 mL of a saturated NaCl solution was added and the samples were gently homogenized and centrifuged at 3000×*g* for 3 min The organic phase was extracted, dried, re-suspended in 250 µL of hexane, and injected into a gas chromatography coupled with a triple quadrupole mass spectrometer (GC–MS Agilent 7890A GC System, Agilent Technologies Inc., Santa Clara, USA)^15^. Fatty acids were separated on a fused silica capillary column (J&W DB-23 Agilent Inc. Santa Clara, USA) with 60 m × 0.250 mm dimensions. Injection volume was 1 μL in the splitless mode and the GC inlet temperature was 250 °C. High-purity Helium was used as the carrier gas at a flow rate of 1.3 mL/min. The oven temperature was programmed to rise from 80 to 175 °C at a rate of 5 °C/min, followed by another gradient of 3 °C/min until 230 °C, which was maintained for 5 min. The transfer line temperature was 280 °C. All mass spectra were obtained by electron impact (70 eV), in the scan mode (40–500 m/z). Compounds were identified by comparing of the retention time of fatty acids in the samples with the retention time of standards (FAME 37 Component Mix Supelco 47885), and also based on a comparison of their mass spectra with those given in the spectral database of the National Institute of Standards and Technology (NIST, Gaithersburg, MD, USA). In case that no evident peak could be observed after integration, it was attribute zero as value. One analysis was carried out/patient and the ratio of the fatty acid/IS area was applied to calculate the percentage of each fatty acid.

### Determination of malondialdehyde (MDA)

MDA concentration was determined by reverse phase HPLC^16^. Briefly, 50 µL of human plasma were mixed with 12.5 μL of 0.2% BHT and 6.25 μL of 10 N NaOH. The TBA–MDA conjugate derivative was injected in a Phenomenex reverse-phase C18 analytical column (250 mm × 4.6 mm; 5 mm; Phenomenex) with an LC8-D8 pre-column (Phenomenex AJ0-1287) coupled to a HPLC (Agilent Technologies 1200 Series). Samples were quantified by fluorometry at an excitation of 515 nm and emission of 553 nm. The HPLC pump delivered the isocratic mobile phase: 40% PBS (A) (10 mmol, pH 7.1) and 60% methanol (B) at a flow rate of 1 mL/min. The gradient applied was: 0–4 min, 40% of solvent A and 60% of solvent B; 4–6 min, 45% of solvent A and 55% of solvent B, and then, the initial conditions were reestablished after 5 min. A standard curve was prepared using 1,1,3,3-Tetraethoxypropane (TEP, T9889 Sigma-Aldrich) (0.10 to 19.97 μmol/L MDA). One analysis was carried out/patient and the concentration was expressed as µmol/L plasma.

### Statistical analysis

According to our hypothesis, the concentration of the most important oxylipin prostaglandin E_2_ (PGE_2_) was chosen for the sample size calculation. Thus, in this context, a sample size of 180 patients was estimated to be enough to have 90% power to detect a difference of 10%, considering a 2-sided alpha of 0.05, based on PGE_2_ mean and deviation reported in the other study^17^. Two statistical approaches were applied to treat these data. Firstly, the characteristics and biomarkers were compared between the five groups using one-way ANOVA, followed by the Tukey test when the values presented a normal distribution (Anderson–Darling test) and homogeneity of variances (Hartley test). Non-parametric Kruskal–Wallis test was followed by controlled comparisons using Bonferroni analysis when the normality and homogeneity assumptions were not verified. For variable “EPA/AA ratio”, Group 5 data was excluded from the statistical analysis, because zero was attributed as value. The analysis of this variable was also adjusted to sex, age and time since diagnosis using gamma distribution. In the second approach, a multivariate analysis was employed as an exploratory tool to identify standards-based characteristics associated with the disease’s severity. From the original continuous variables, 22 were selected to be included as active variables in the multivariate analysis. The criteria applied to this selection was to change according to the five groups. The principal component analysis (PCA) was based on correlation. Adopting Ward’s method and Euclidean distance, cluster analysis was carried out to group variables and patients. The significance was set at a p-value of 0.05. Analyses and graphs were performed using Statistica v. 13.4 (TIBCO Software Inc, Round Rode, Texas, USA), GraphPad Prism 9.0 and R v. 4.0.4 (R Development Core Team, 2021).

## Results

### Population

The general patient characteristics at hospital admission (Table 1) showed a wide range of age and BMI. The biomarkers determined in the patients at hospital admission are shown in Table 2. Furthermore, the oxidative stress biomarkers indicated large variability between the patients. Drugs prescribed to less than 5% of the patients during the hospital stay were described in Supplementary Table 1.

**Table 1:** General patient characteristics at hospital admission.

Characteristics	n	Mean (± SEM)	Median	Range (min–max)
Anthropometric data				
Age (years)	180	55.34 (± 1.09)	56.50	20.00–87.00
Weight (kg)	173	85.57 (± 1.55)	85.00	42.00–175.00
Height (m)	167	1.67 (± 0.01)	1.66	1.45–2.00
BMI (kg/m^2^)	167	30.94 (± 0.51)	30.12	17.91–62.50
Hospital admission				
Days from the first symptoms (days)	180	10.12 (± 0.28)	10.00	2.00–23.00
Hospital length of stay (days)	174	7.02 (± 0.56)	5.00	0.00–49.00
Orotracheal intubation (days)	175	1.03 (± 0.33)	0.00	0.00–30.00
Laboratory data				
Urea nitrogen (mg/dL)	179	38.84 (± 0.97)	38.00	13.00–87.00
Creatinine (mg/dL)	180	0.84 (± 0.02)	0.82	0.42–1.72
C-reactive protein (mg/dL)	180	68.88 (± 5.21)	51.00	1.60–397.20
RBC (10^12^/L)	180	4.66 (± 0.05)	4.67	2.39–6.71
Hemoglobin (g/dL)	180	13.44 (± 0.14)	13.50	7.40–17.70
Platelet (10^3^/µL)	180	292.47 (± 9.05)	276.50	38.00–723.00
D-dimer (mg/L)	178	1605.72 (± 356.50)	758.50	190.00–57,811.00
Leukocytes (10^3^/µL)	180	9.18 (± 0.26)	8.56	1.80–20.87
Lymphocyte (10^3^/µL)	180	1.27 (± 0.09)	1.02	0.19–15.00
Neutrophils (10^3^/µL)	180	7.29 (± 0.24)	6.85	1.06–17.49
Eosinophils (10^3^/µL)	179	0.04 (± 0.01)	0.00	0.00–0.56
Erythrocyte sedimentation rate (mm/h)	176	48.46 (± 2.80)	46.50	1.00–140.00
Total cholesterol (mg/dL)	177	171.16 (± 3.33)	169.00	65.00–301.00
High density lipoprotein (mg/dL)	176	36.66 (± 0.82)	36.00	18.00–93.00
Low density lipoprotein (mg/dL)	176	104.59 (± 2.72)	102.00	25.00–206.00
Triacylglycerol (mg/dL)	176	187.30 (± 6.09)	179.50	49.00–526.00

	%			
Sociodemographic data				
Sex				
Female	46.67			
Male	53.33			
Ethinicity				
White	44.78			
Pardo^a^	38.33			
Black	13.89			
Comorbidities				
Obesity	53.29			
Hypertension	46.67			
Chronic heart disease	11.67			
Chronic obstructive pulmonary disease (COPD)	3.89			
Asthma	5.56			
Diabetes	25.56			
Diarrhea	47.22			
Rheumatic diseases	8.33			
Others	41.11			
Main pharmacological treatment during the hospital stay^b^				
Acetylsalicylicacid (100 mg)	7.22			
Albuterol (100 mg)	18.33			
Amlodipine (5 mg)	12.22			
Atenolol (50 mg)	8.33			
Azithromycin (500 g)	57.22			
Captopril (25 mg)	10.56			
Ceftriaxone (1 g)	84.44			
Clonazepam (2.5 mg/mL)	8.89			
Codeine Sulfate (5 mg)	10.00			
Dexamethasone (6 mg)	75.0			
Dipyrone (1 g)	5.00			
Dipyrone (500 mg)	58.33			
Enalapril (20 mg)	5.00			
Enoxaparin (40 mg)	87.78			
Hydrochlorothiazide (25 mg)	6.11			
Hydrochlorothiazide (50 mg)	6.67			
Lactulose (667 mg)	9.44			
Levothyroxine (50 µg)	5.00			
Losartan (50 mg)	30.56			
Metformin (850 mg)	9.44			
Metoclopramide (5 mg)	51.11			
NPH insulin (25 25 25)	8.89			
Omeprazole (20 mg)	56.11			
Omeprazole (40 mg)	5.56			
Ondansetron (2 mg)	16.67			
Simvastatin (40 mg)	11.67			
Hospital admission				
Oxygen supply	60.00			
High-flow nasal canula (HFNC) oxygen, noninvasive ventilation (NIV)	10.00			
Orotracheal intubation + invasive ventilation (NIV)	9.44			
Intensive care unit (ICU)	14.44			
Clinical end-points (discharge, death, dropout)	93.33, 3.89, 2.78			

SI conversion factors: To convert creatinine to μmol/L, multiply values by 88.4; D-dimer to nmol/L, multiply values by 5.476.

^a^Pardo is the exact term used in Brazilian Portuguese, meaning “mixed ethnicity”, according to the Brazilian Institute of Geography and Statistics.

^b^Drugs that were prescribed to less than 5% of the patients during the hospital stay were described in Supplementary Table 1.

**Table 2:** Fatty acids, oxidative stress and inflammatory biomarkers of the patients at hospital admission.

	n	Mean (± SEM)	Median	Range (min–max)
Fatty acids (%)				
C12:0 (lauric acid)	180	0.10 (± 0.01)	0.00	0.00–0.68
C14:0 (myristic acid)	180	0.40 (± 0.01)	0.39	0.00–0.98
C16:0 (palmitic acid)	180	37.87 (± 0.24)	37.97	20.62–47.22
16:1 n7(palmitoleic acid)	180	1.06 (± 0.03)	0.96	0.32–3.08
17:0 (heptadecanoic acid)	180	0.36 (± 0.01)	0.32	0.00–0.68
18:0 (stearic acid)	180	27.65 (± 0.44)	28.13	0.00–40.54
18:1 n9 (oleic acid)	180	11.96 (± 0.25)	11.23	1.47–24.20
18:2 n6 (linoleic acid)	180	15.11 (± 0.39)	14.95	0.00–29.81
18:3 n6 (γ-linolenic acid)	180	0.10 (± 0.01)	0.00	0.00–0.73
18:3 n3 (α-linolenic acid)	180	0.15 (± 0.01)	0.00	0.00–0.75
20:0 (arachidic acid)	180	0.06 (± 0.01)	0.00	0.00–0.43
20:2 n6 (eicosadienoic acid)	180	0.06 (± 0.01)	0.00	0.00–0.51
20:3 n6(dihomo-γ-linolenicacid)	180	0.56 (± 0.02)	0.54	0.00–1.68
20:4 n6 (arachidonic acid)	180	3.79 (± 0.09)	3.69	1.58–8.45
20:5 n3 (eicosapentaenoic acid)	180	0.15 (± 0.02)	0.10	0.00–1.19
22:6 n3 (docosahexaenoic acid)	180	0.54 (± 0.02)	0.51	0.00–1.43
SFA	180	66.45 (± 0.62)	67.08	39.29–85.27
MUFA	180	13.01 (± 0.27)	12.30	2.92–27.29
PUFA	180	20.55 (± 0.44)	20.34	3.33–36.78
Omega 3 FA	180	0.93 (± 0.03)	0.90	0.00–2.24
Omega 6 FA	180	19.61 (± 0.43)	19.28	2.95–35.82
n-3/n-6 FA ratio	180	0.05 (± 0.003)	0.05	0.00–0.32
(EPA + DHA)/AA ratio	180	0.18 (± 0.01)	0.17	0.00–0.56
EPA/AA ratio	180	0.04 (± 0.004)	0.03	0.00–0.22
Oxidative stress				
MDA (µmol)	180	4.78 (± 0.20)	4.14	1.39–19.76
PGE_2_ (ng/mL)	180	0.68 (± 0.06)	0.47	0.00–8.02
15-HETE (ng/mL)	180	3.16 (± 0.14)	2.82	0.00–11.64
12-HETE (ng/mL)	180	12.04 (± 6.47)	1.34	0.00–870.78
5-HETE (ng/mL)	180	2.02 (± 0.14)	1.60	0.00–12.89
12,13-DiHOME (ng/mL)	180	4.34 (± 0.29)	3.70	0.00–25.86
9,10-DiHOME (ng/mL)	180	1.96 (± 0.13)	1.49	0.00–13.27
12,13-EpOME (ng/mL)	180	13.78 (± 0.65)	11.63	0.00–65.60
9,10-EpOME (ng/mL)	180	31.57 (± 1.65)	23.88	7.81–174.36
13-HODE (ng/mL)	180	9.37 (± 0.47)	7.22	2.10–46.53
14,15-EET (ng/mL)	180	2.55 (± 0.14)	2.02	0.00–12.94
11,12-EET (ng/mL)	180	18.58 (± 1.22)	14.42	2.92–178.33
5-series-F_2_-IsoP (ng/mL)	180	6.41 (± 0.08)	6.40	3.98–9.91
Cytokines				
IL-1β (pg/mL)	178	2.00 (± 0.24)	0.94	0.01–23.56
IL-6 (pg/mL)	178	19.05 (± 2.80)	4.53	0.19–276.00
TNFα (pg/mL)	178	9.62 (± 0.64)	7.29	0.86–54.84

*SFA* saturated fatty acids, *MUFA* monoinsaturated fatty acids, *PUFA* polyunsaturated fatty acids, *5-HETE* 5-hydroxyeicosatetraenoic acid, *5-series-F*_*2*_*-IsoP* 5-series F_2_-isoprostanes, *12-HETE* 12-hydroxyeicosatetraenoic acid, *9,10-diHOME 9,10*-dihydroxy-12-octadecenoic acid, *12,13-diHOME* 12,13-dihydroxy-9Z-octadecenoic acid, *9,10 EpOME* 9,10-epoxy-12Z-octadecenoic acid, *12,13 EpOME* 12,13-Epoxy-9(Z)-octadecenoic acid, *13-HODE* 13-hydroxyoctadecadienoic acid, *11,12-EET* 11,12-epoxyeicosatrienoic acid, *14,15-EET* 14,15-epoxyeicosa-5.8.11-trienoic Acid, *15-HETE* 15-hydroxyeicosatetraenoic acid, *AA* arachidonic acid, *DHA* docosahexaenoic acid, *EPA* eicosapentaenoic acid, *IL-1β* interleukin 1β, *IL-6* interleukin 6, *MDA* malondialdehyde, *PGE*_*2*_ prostaglandin E_2_, *TNF α* tumor necrosis factor α.

### Biomarkers according to disease severity during hospitalization

**Figure 1:**
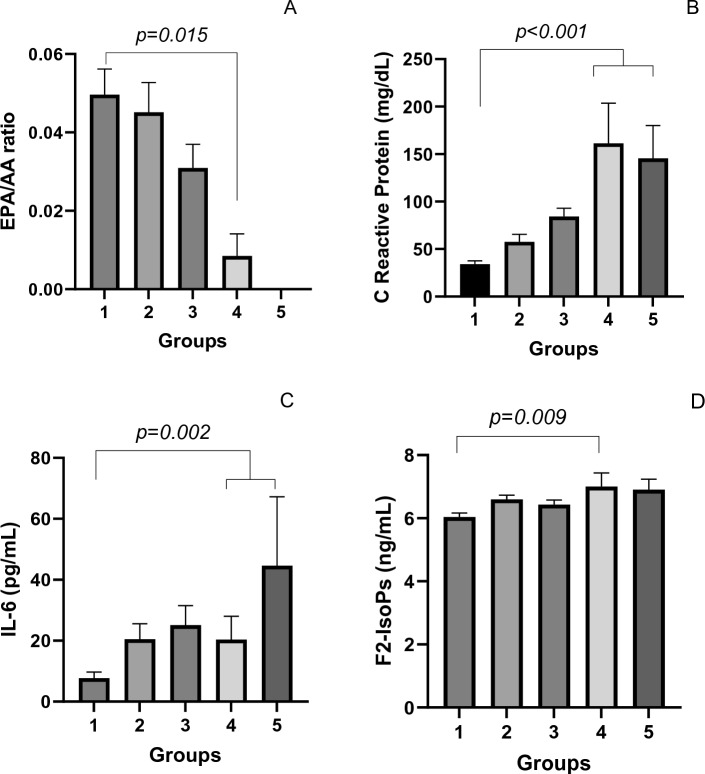
Plasma fatty acids, cytokines and 5-series-F_2_-IsoP concentration presented by the patients classified into 5 groups. Mass spectrometry analysis of plasma fatty acids, cytokines and 5-series-F_2_-IsoP concentration according to the disease severity that increased from Group 1 to Group 5. (**A**) EPA/AA ratio; (**B**) C-reactive protein (CRP) (mg/dL); (**C**) Interleukin-6 (IL-6) (pg/mL); (**D**) 5-series-F_2_-IsoP concentration (ng/mL). Boxes and whiskers represent mean and SEM respectively. P-values were obtained by One-way ANOVA followed by Tukey HSD test or equivalent non-parametric Kruskal Wallis Test followed by Bonferroni corrections, both for independent groups (n: 177–179).

Anthropometric data, immune cells, oxylipins and cytokines concentration, and fatty acids proportion determined in the patient’s plasma classified according to the five groups are shown in Table 3. From these values, the most relevant results associated with our hypothesis that showed different values between Groups 4 and/or 5 and Groups 1, 2, and 3 are presented in Fig. 1. Although no difference has been observed to the n-3FA/n-6FA ratio among the groups (*p* = 0.276), the EPA/AA ratio decreased from Group 1 to Group 4 (*p* = 0.015; Fig. 1A). The EPA/AA ratio was adjusted for sex, age and time since diagnosis, being just this later factor associated to the severity of the disease (*p* = 0.025) (Supplementary Table 2). Subsequently, this result was followed by two inflammatory markers in COVID-19 patients: C-reactive protein (Fig. 1B) and Interleukin-6 (Fig. 1C). Concerning F_2_-Isop concentration, Group 4 had higher values in comparison to Group 1 (Fig. 1D). All these biomarkers showed correlation with the severity of the disease.

**Table 3:** Characteristics of the patients determined at hospital admission (baseline) according to the stratification based on the disease severity.

Characteristics	Groups of patients (n = 179^Ɨ^)					p-value*
	GROUP 1 (n = 53)	GROUP 2 (n = 51)	GROUP 3 (n = 58)	GROUP 4 (n = 10)	GROUP 5 (n = 7)	
Anthropometric data						
Age (years)	49.94 (± 1.86)^a^	53.98 (± 2.02)^ab^	59.57 (± 1.88)^b^	57.20 (± 4.56)^ab^	66.43 (± 5.39)^b^	0.002
BMI (kg/m^2^)	31.00 (± 0.85)^a^	31.38 (± 0.72)^a^	30.95 (± 1.13)^a^	34.23 (± 2.03)^a^	22.75 (± 0.66)^b^	0.005
Laboratory data						
Urea nitrogen (mg/dL)	38.34 (± 1.42)	35.61 (± 1.84)	41.00 (± 1.75)	38.78 (± 2.32)	45.29 (± 9.13)	0.089
Creatinine (mg/dL)	0.85 (± 0.03)	0.82 (± 0.03)	0.84 (± 0.03)	0.87 (± 0.11)	0.87 (± 0.11)	0.908
C-reactive protein (mg/dL)	33.98 (± 3.60)^a^	57.65 (± 7.86)^ab^	84.25 (± 8.74)^bc^	161.33 (± 42.31)^bc^	145.44 (± 34.63)^c^	< 0.001
RBC (10^12^/L)	4.86 (± 0.08)^a^	4.45 (± 0.10)^b^	4.59 (± 0.07)^ab^	5.12 (± 0.23)^a^	4.63 (± 0.23)^ab^	0.002
Hemoglobin (g/dL)	14.03 (± 0.23)^a^	12.81 (± 0.29)^b^	13.37 (± 0.20)^ab^	14.18 (± 0.58)^ab^	13.23 (± 0.65)^ab^	0.020
Platelet (10^3^/µL)	308.15 (± 15.15)	298.90 (± 19.78)	291.40 (± 15.47)	226.30 (± 26.60)	248.57 (± 38.62)	0.172
D-dimer (mg/L)	1999.06 (± 1079.31)^ab^	1035.1 (± 141.69)^ab^	1974.91 (± 466.25)^a^	452.56 (± 75.39)^b^	1382.71 (± 247.00)^a^	0.007
Leukocytes (10^3^/µL)	8.72 (± 0.35)	8.59 (± 0.50)	9.90 (± 0.54)	10.17 (± 1.05)	9.32 (± 1.25)	0.231
Lymphocyte (10^3^/µL)	1.50 (± 0.11)^a^	1.43 (± 0.28)^ab^	0.97 (± 0.05)^b^	1.51 (± 0.59)^ab^	0.67 (± 0.14)^b^	< 0.001
Neutrophils (10^3^/µL)	6.54 (± 0.34)^a^	6.45 (± 0.40)^a^	8.42 (± 0.52)^b^	8.13 (± 0.75)^ab^	8.17 (± 1.25)^ab^	0.014
Eosinophils (10^3^/µL)	0.04 (± 0.01)	0.05 (± 0.01)	0.03 (± 0.01)	0.05 (± 0.05)	0.01 (± 0.01)	0.099
Erythrocyte sedimentation rate (mm/h)	41.95 (± 4.97)	45.20 (± 5.66)	55.66 (± 4.58)	50.70 (± 11.40)	57.90 (± 18.50)	0.228
Total cholesterol (mg/dL)	183.38 (± 5.42)	172.22 (± 5.92)	161.19 (± 6.73)	161.30 (± 11.84)	174.17 (± 12.13)	0.112
High density lipoprotein (mg/dL)	37.00 (± 1.18)	36.64 (± 1.26)	36.56 (± 1.94)	37.78 (± 2.81)	33.67 (± 3.52)	0.643
Low density lipoprotein (mg/dL)	113.87 (± 4.38)	105.96 (± 4.96)	96.56 (± 5.38)	92.11 (± 9.02)	112.67 (± 12.71)	0.099
Triacylglycerol (mg/dL)	204.45 (± 11.13)^a^	197.46 (± 10.90)^ab^	173.46 (± 11.40)^ab^	142.33 (± 19.13)^b^	163.50 (± 15.25)^ab^	0.013
Fatty acids (%)						
C12:0 (lauric acid)	0.14 (± 0.02)^a^	0.09 (± 0.02)^ab^	0.09 (± 0.02)^b^	0.07 (± 0.05)^ab^	0.02 (± 0.02)^ab^	0.039
C14:0 (myristic acid)	0.46 (± 0.03)	0.37 (± 0.03)	0.38 (± 0.02)	0.43 (± 0.04)	0.40 (± 0.04)	0.138
C16:0 (palmitic acid)	37.69 (± 0.46)	37.72 (± 0.33)	37.93 (± 0.50)	39.37 (± 0.97)	37.28 (± 0.63)	0.629
C16:1 n7 (palmitoleic acid)	1.16 (± 0.07)^a^	1.05 (± 0.05)^ab^	1.05 (± 0.06)^ab^	0.78 (± 0.07)^b^	0.74 (± 0.09)^ab^	0.033
C17:0 (margaric acid)	0.35 (± 0.02)^a^	0.34 (± 0.02)^a^	0.35 (± 0.02)^a^	0.50 (± 0.04)^b^	0.43 (± 0.06)^ab^	0.021
C18:0 (stearic acid)	27.17 (± 0.97)	27.92 (± 0.77)	27.76 (± 0.73)	28.36 (± 1.26)	26.71 (± 1.19)	0.970
C18:1 n9 (oleic acid)	12.30 (± 0.51)	11.63 (± 0.42)	12.06 (± 0.46)	11.42 (± 0.66)	12.24 (± 0.88)	0.908
C18:2 n6 (linoleic acid)	15.08 (± 0.86)	15.53 (± 0.54)	14.93 (± 0.72)	13.37 (± 1.69)	16.71 (± 1.25)	0.759
C18:3 n6 (γ linolenic acid)	0.13 (± 0.02)^a^	0.12 (± 0.02)^ab^	0.08 (± 0.02)^ab^	0.00 (± 0.00)^b^	0.03 (± 0.03)^ab^	0.008
C18:3 n3 (α-linolenic acid)	0.21 (± 0.03)	0.16 (± 0.03)	0.10 (± 0.02)	0.11 (± 0.04)	0.16 (± 0.06)	0.132
C20:0 (arachidic acid)	0.07 (± 0.02)	0.05 (± 0.01)	0.06 (± 0.01)	0.02 (± 0.02)	0.05 (± 0.04)	0.577
C20:2 n6 (eicosadienoicacid)	0.07 (± 0.02)	0.05 (± 0.01)	0.06 (± 0.01)	0.06 (± 0.04)	0.03 (± 0.03)	0.841
C20:3 n6 (dihomo-γ-linolenicacid)	0.63 (± 0.04)	0.51 (± 0.04)	0.53 (± 0.05)	0.74 (± 0.08)	0.59 (± 0.08)	0.064
C20:3 n3	0.12 (± 0.02)	0.08 (± 0.02)	0.09 (± 0.02)	0.08 (± 0.04)	0.04 (± 0.04)	0.064
C20:4 n6 (arachidonic acid)	3.70 (± 0.17)	3.70 (± 0.14)	3.89 (± 0.17)	4.00 (± 0.21)	3.92 (± 0.22)	0.664
C20:5 n3 (eicosapentaenoicacid)	0.19 (± 0.03)	0.18 (± 0.03)	0.13 (± 0.02)	0.04 (± 0.02)	nd	0.032
C22:6 n3 (docosahexaenoic acid)	0.54 (± 0.04)	0.50 (± 0.03)	0.53 (± 0.03)	0.66 (± 0.07)	0.65 (± 0.14)	0.301
SAFA	65.87 (± 1.27)	66.50 (± 1.05)	66.56 (± 1.16)	68.75 (± 2.10)	64.89 (± 1.70)	0.869
MUFA	13.46 (± 0.57)	12.68 (± 0.46)	13.11 (± 0.49)	12.20 (± 0.72)	12.98 (± 0.96)	0.859
PUFA	20.67 (± 0.92)	20.83 (± 0.67)	20.32 (± 0.81)	19.05 (± 1.88)	22.13 (± 1.33)	0.850
Omega 3 FA	1.06 (± 0.07)	0.92 (± 0.05)	0.85 (± 0.06)	0.89 (± 0.10)	0.85 (± 0.11)	0.171
Omega 6 FA	19.62 (± 0.91)	19.91 (± 0.64)	19.48 (± 0.79)	18.17 (± 1.86)	21.29 (± 1.34)	0.845
Omega 3/omega 6 ratio	0.06 (± 0.01)	0.05 (± 0.002)	0.05 (± 0.003)	0.07 (± 0.02)	0.04 (± 0.01)	0.276
EPA + DHA/AA	0.20 (± 0.01)	0.18 (± 0.01)	0.17 (± 0.01)	0.18 (± 0.02)	0.16 (± 0.03)	0.327
EPA/AA ratio	0.05 (± 0.01)^a^	0.04 (± 0.01)^ab^	0.03 (± 0.01)^ab^	0.01 (± 0.01)^b^	nd	0.015
Oxylipins						
MDA (µmol)	4.08 (± 0.18)^a^	4.08 (± 0.23)^a^	5.81 (± 0.45)^b^	4.79 (± 0.82)^ab^	6.11 (± 1.88)^ab^	0.009
PGE2 (ng/mL)	0.96 (± 0.09)^a^	0.46 (± 0.06)^b^	0.69 (± 0.15)^ab^	0.41 (± 0.08)^b^	0.63 (± 0.17)^ab^	< 0.001
15-HETE (ng/mL)	2.46 (± 0.18)^a^	3.42 (± 0.27)^b^	3.68 (± 0.28)^b^	2.17 (± 0.34)^ab^	3.60 (± 0.67)^b^	0.002
12-HETE (ng/mL)	3.36 (± 1.17)	4.44 (± 2.70)	29.95 (± 19.81)	1.12 (± 0.29)	2.08 (± 0.55)	0.483
5-HETE (ng/mL)	1.93 (± 0.29)	2.14 (± 0.23)	2.20 (± 0.28)	1.28 (± 0.25)	1.38 (± 0.47)	0.489
12,13-DiHOME (ng/mL)	5.73 (± 0.52)^a^	3.43 (± 0.41)^b^	4.18 (± 0.64)^b^	2.81 (± 0.61)^b^	4.22 (± 0.93)^ab^	< 0.001
9,10-DiHOME (ng/mL)	2.62 (± 0.33)^a^	1.62 (± 0.14)^ab^	1.81 (± 0.23)^b^	1.25 (± 0.27)^ab^	1.73 (± 0.45)^ab^	0.033
12,13-EpOME (ng/mL)	12.89 (± 0.79)	14.39 (± 1.04)	14.84 (± 1.48)	7.91 (± 1.28)	16.36 (± 5.40)	0.095
9,10-EpOME (ng/mL)	27.38 (± 1.73)	32.20 (± 2.45)	35.76 (± 3.89)	20.97 (± 2.26)	41.55 (± 15.52)	0.428
13-HODE (ng/mL)	8.63 (± 0.53)	9.70 (± 0.74)	10.21 (± 1.08)	5.55 (± 0.60)	11.47 (± 4.11)	0.067
14,15-EET (ng/mL)	2.21 (± 0.21)	2.85 (± 0.25)	2.52 (± 0.22)	2.09 (± 0.26)	3.96 (± 1.59)	0.273
11,12-EET (ng/mL)	16.88 (± 1.13)	19.17 (± 1.53)	20.75 (± 3.13)	10.84 (± 1.90)	21.69 (± 10.79)	0.058
5-Series-F_2_-IsoP (ng/mL)	6.04 (± 0.13)^a^	6.59 (± 0.13)^b^	6.43 (± 0.14)^ab^	7.00 (± 0.43)^b^	6.90 (± 0.34)^ab^	0.009
Cytokines						
IL-1β (pg/mL)	1.78 (± 0.33)	2.14 (± 0.49)	2.13 (± 0.46)	2.74 (± 1.49)	0.74 (± 0.15)	0.330
IL-6 (pg/mL)	7.73 (± 2.00)^a^	20.47 (± 5.08)^ab^	25.13 (± 6.35)^ab^	20.39 (± 7.62)^b^	44.65 (± 22.58)^b^	0.002
TNF-α (pg/mL)	9.02 (± 1.03)	9.18 (± 1.11)	10.54 (± 1.32)	11.54 (± 2.85)	7.38 (± 2.47)	0.674
Hospital admission						
Days from the firsts symptoms (days)	10.62 (± 0.53)	11.29 (± 0.41)	9.22 (± 0.49)	8.30 (± 1.44)	8.57 (± 1.11)	–
Hospital length of stay (days)	3.38 (± 0.37)	3.98 (± 0.13)	8.18 (± 0.43)	31.14 (± 3.97)	22.57 (± 4.05)	–
Orotracheal intubation (days)	–	–	–	9.86 (± 1.44)	18.50 (± 4.40)	–
Sociodemographic data						
Sex						
Male (%)	57	39	59	50	86	0.095
Ethnicity (%)						0.501
White	45	51	52	40	29	
Pardo	43	37	31	30	71	
Black	12	12	17	30	–	
Comorbidities						
Obesity	53	58	51	88	–	0.021
Hypertension	45	39	55	30	57	0.375
Chronic heart disease	6	12	12	20	29	0.215
COPD	–	2	10	–	–	0.075
Asthma	2	8	7	10	–	0.477
Diabetes	21	18	34	30	29	0.269
Diarrhea	43	51	47	50	43	0.949
Rheumatic diseases	11	10	5	10	–	0.695

*5-HETE* 5-hydroxyeicosatetraenoic acid, *5-series-F*_*2*_*-IsoP* 5-series F_2_-isoprostanes, *12-HETE* 12-Hydroxyeicosatetraenoic acid, *9,10-diHOME 9,10*-dihydroxy-12-octadecenoic acid, *12,13-diHOME* 12,13-dihydroxy-9Z-octadecenoic acid, *9,10 EpOME* 9,10-epoxy-12Z-octadecenoic acid, *12,13 EpOME* 12,13-epoxy-9(Z)-octadecenoic acid, *13-HODE* 13-Hydroxyoctadecadienoic acid, *11,12-EET* 11,12-epoxyeicosatrienoic acid, *14,15-EET* 14,15-epoxyeicosa-5.8.11-trienoic acid, *15-HETE* 15-hydroxyeicosatetraenoic acid, *COPD* chronic obstructive pulmonary disease, *nd* not detected.

*Values were expressed as mean (± SEM). p-values were obtained by ANOVA or Kruskal Wallis Test for quantitative variables and by Fisher Test for categorized variables. Variables C20:5 n3 (eicosapentaenoicacid) and EPA/DHA ratio was evaluated considering only Groups 1 to 4. Values followed by the same letter are not different (p < 0.05).

^Ɨ^The number of sample/group can change according to the analyzed parameter.

### Biomarkers according to the cluster analysis

**Figure 2:**
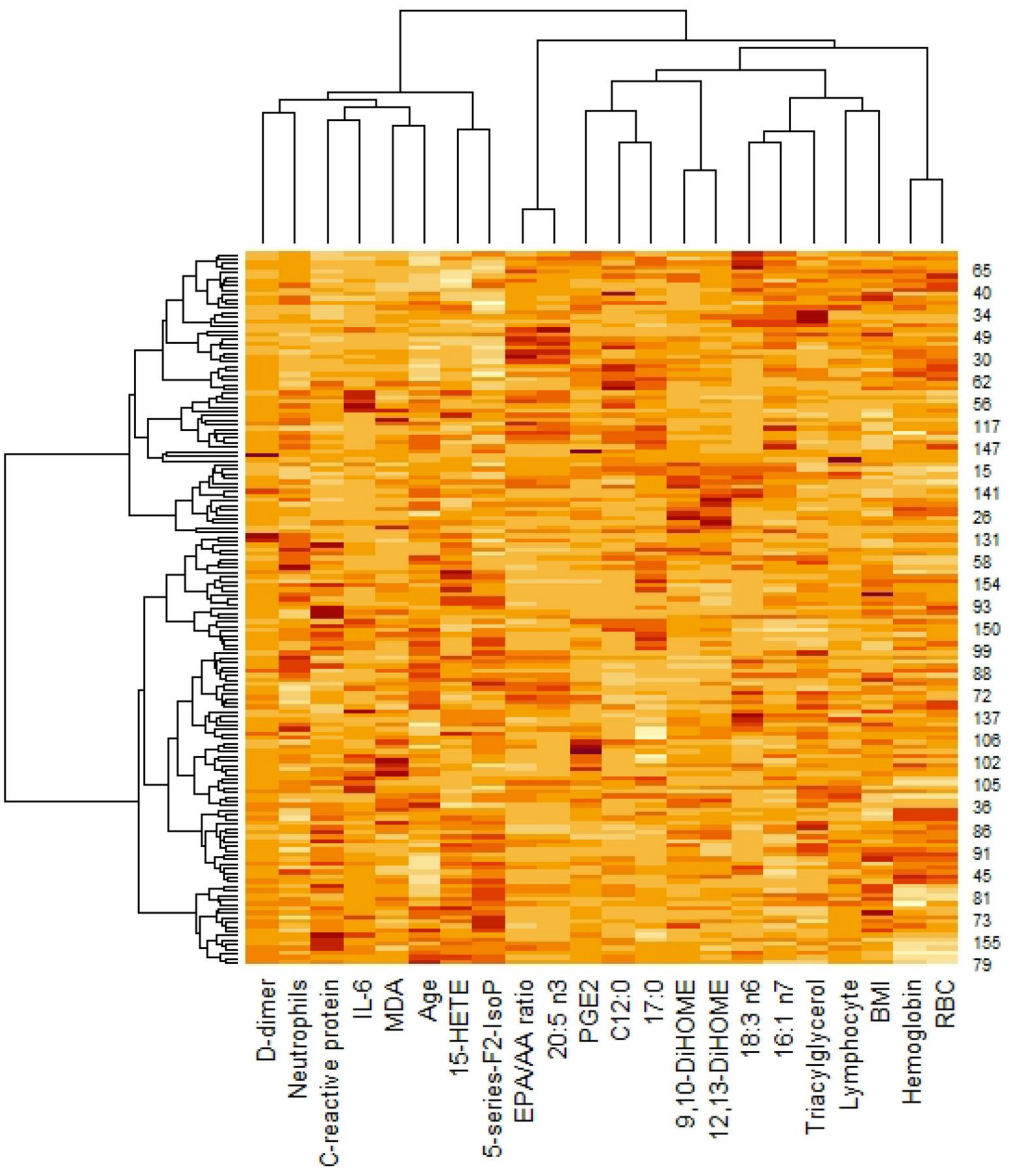
Heatmap of the patients according to the selected biomarkers. Heatmap representing the patients (159) according to the cluster analysis using Ward’s method (22 variables). Rows represent individual values observed for each variable (columns), including the dendrogram obtained to patients and variables. Figure was made using the software R version 4.04 URL http://www.R-project.org (R Core Team. R: A language and environment for statistical computing. Vienna: R Foundation for Statistical Computing, 2014).

A multivariate analysis that considered 22 biomarkers was performed using another statistical approach. The original matrix was standardized according to the mean and deviation and applied to the cluster analysis using Ward’s method and Euclidean distance. Figure 2 shows the patients’ heatmap. All levels of severity were distributed among the clusters, suggesting that there was no standard of reclassification of the patients. This result was confirmed through a discriminant analysis (Supplementary Table 3), in which the rate of success of the patient’s classification based on the 10 major PCs was only 42.80%.

## Discussion

Initially, the general characteristics of the patients, as shown in Table 1, were consistent with the majority of data concerning other SARS-CoV-2 patients hospitalized in 2020, considering that no vaccines were viable, and drugs were administered only to alleviate the symptoms and avoid other infections^5,7,18,19^. However, the BMI of Group 5 was unexpected, as the most severe conditions were typically associated with overweight and obesity^20^. In this regard, the intense catabolic state induced by SARS-CoV-2 infection results in significant body weight loss, primarily characterized by a reduction in lean mass. Consequently, it becomes feasible to contemplate a higher prevalence of sarcopenia among patients in Group 5.

The primary challenge in this study was to detect a difference in the n-3 FA/n-6 FA ratio among the patients, given the known the low n-3 FA supplements or fish consumption in the Western diet^21^. Thus, the difference observed in the EPA concentration could have been due to the endogenous conversion from α-linolenic acid (ALA), which was also low^3,22^. Although the cellular incorporation of EPA and DHA occurs mainly at the expense of AA^23^, this replacement seems to depend on the fatty acid pool^24^. This could explain the absence of any observed changes in the AA proportion among the patients (*p* = 0.664), especially considering the absence of any n-3 FA supplementation. DHA is known to exhibit a higher plasma concentration than EPA^25^, but its variability is lower compared to EPA^9^ due to the process involving the transfer of EPA to peroxisomes, where it undergoes β-oxidation to form DHA^26^. Furthermore, DHA undergoes rapid incorporation into phospholipids and cholesterol esterification^27^.

**Figure 3:**
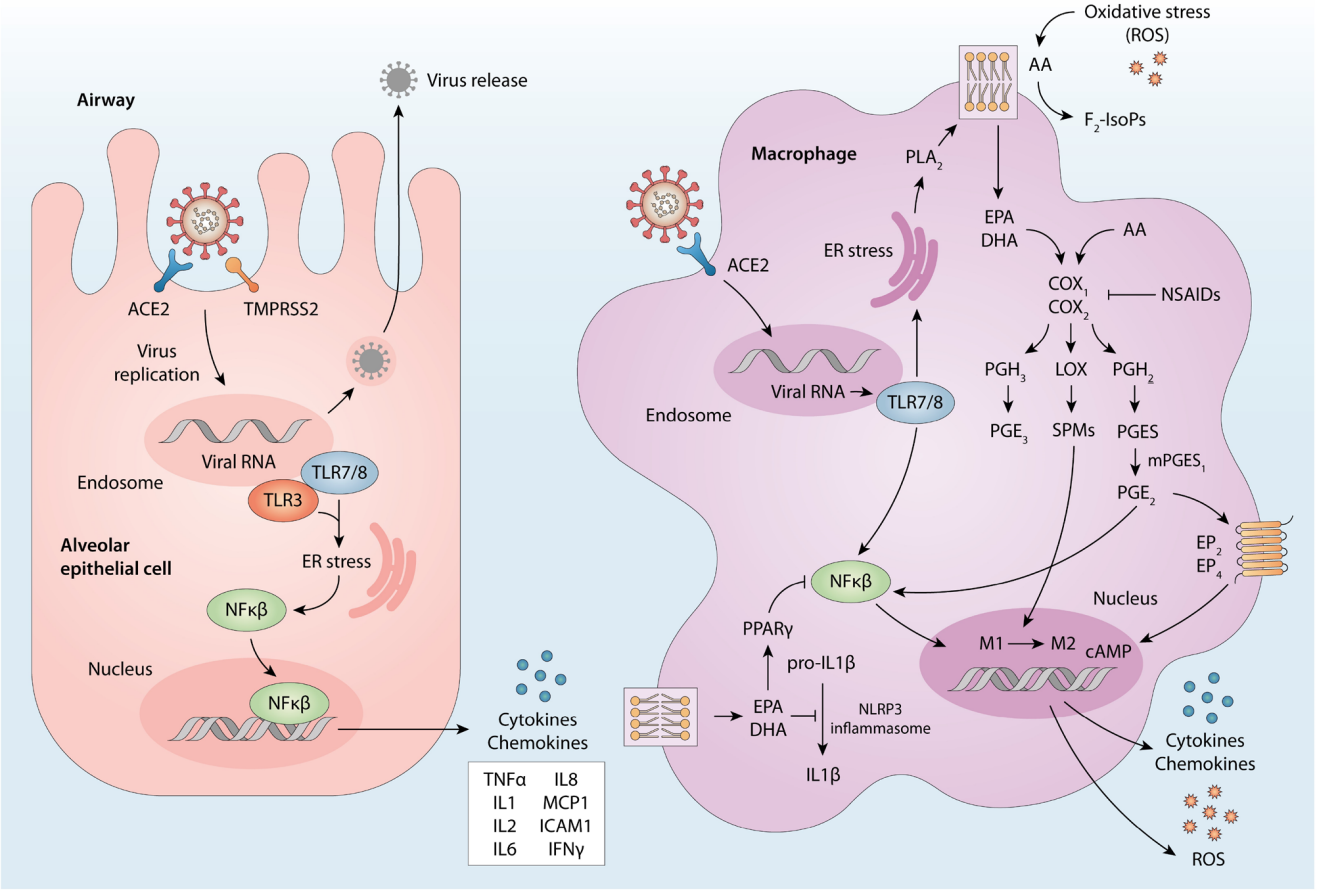
Summary of the main hypothesis of this study. The SARS-CoV-2 virus present in the airway infects cells that express the surface receptors angiotensin-converting enzyme 2 (ACE2) and transmembrane serine protease 2 (TMPRSS2), such as alveolar epithelial cells. The virus’s surface spike protein (S) binds to ACE2, initiating endocytosis mediated by TMPRSS2. Acidification of the endosome triggers viral and cellular membrane fusion, allowing the release of viral single-stranded RNA (ssRNA) into the cytosol. Subsequently, the virus undergoes replication and is released back into the airway. Within the endosomes, viral RNA activates Toll-like receptors (TLR3, TLR7/8), inducing endoplasmic reticulum (ER) stress. This stress leads to the release of NFκB, which translocates to the nucleus, initiating the transcription of genes for inflammatory cytokines, chemokines, adhesion molecules, and growth factors. These proteins attract monocytes and T cells to the infection site, establishing a pro-inflammatory feedback loop. In the macrophages, in response to ER stress, phospholipase A_2_ (PLA_2_) hydrolyzes fatty acids esterifying phospholipids in the membranes, serving as substrates for oxidative enzymes, producing various oxylipins. Arachidonic acid (AA) is converted by cyclooxygenase (COX) into PGH_2_, further transformed into PGE_2_, binding to EP_2_/EP_4_ receptors on macrophage membranes. This activation of cAMP intensifies inflammation, causing pain, immunoregulation, mitogenesis, and cell injury. Conversely, if eicosapentaenoic acid (EPA) and docosahexaenoic acid (DHA) are released, PGH_3_ is synthesized. This mechanism explains the use of NSAIDs in reducing COVID-19 symptoms. Excessive reactive oxygen species (ROS) generated by macrophages can oxidize AA within phospholipids, increasing F_2_-IsoPs concentration. It is possible that EPA and DHA might reduce pro-inflammatory molecules by activating peroxisome proliferator-activated receptor gamma (PPARγ), consequently inhibiting NFκB translocation to the nucleus. Additionally, EPA and DHA may inhibit the NLRP3 inflammasome, impeding interleukin-1β maturation, and induce specialized pro-resolving mediators (SPMs) to alter macrophage phenotypes, facilitating inflammation resolution^2,4,25,34,43,48^. *ACE2* angiotensin-converting enzyme 2, *TMPRSS2* transmembrane serine protease 2, *TLR3, TLR7/8* toll-like receptors, *ER* endoplasmic reticulum, *NFκB* nuclear factor-kappa B, *PLA*_*2*_ phospholipase A_2_, *AA* arachidonic acid, *PGE*_*2*_ prostaglandin E_2_, *COX* cyclooxygenase, *LOX* lipoxygenase, *cAMP* cyclic adenosin monophopshate, *NSAIDs* non-steroidal anti-inflammatory drugs, *F*_*2*_*-IsoPs* F_2_-isoprostanes, *PPARγ* peroxissome proliferator-activated receptor gamma, *NLRP3* NLR family pyrin domain containing 3, *IL-1β* inteleukin 1β, *SPMs* specialized pro-resolving mediators, *EPA* eicosapentaenoic acid, *DHA* docosahexaenoic acid.

The main hypothesis of this study is summarized in Fig. 3. It is supposed that after SARS-CoV-2 infection, the innate immune system cells promote the synthesis of cytokines, chemokines, growth factors, and adhesion molecules, trying to counteract virus replication and spreadability. In addition, PLA_2_, among other hydrolases, releases fatty acids from the phospholipid chain to be used as a substrate for oxidant enzymes, such as lipoxygenases (LOX), cyclooxygenases (COX), and cytochrome P450 (CytP450), leading to the formation of many oxylipins involved in the immune response and the resolution of inflammation^26,28^. It has been reported that EPA- and DHA-derived oxylipins have a less potent inflammatory action than AA-derived oxylipins^26^. For example, PGE_2_ formed from AA (Fig. 3) has been associated with increased cytokine expression, although this effect depends on several other conditions^9^.

Our results clearly showed that a lower EPA/AA ratio, mainly observed in Groups 4–5 (Fig. 1A), was associated with a higher concentration of IL-6 and CRP, typically elevated in critically ill COVID-19 patients^4,6,29,30^, bringing a worse prognostic to these patients. In a retrospective pilot study^31^, it was observed an increase in maresin (MaR2) in severe COVID-19 disease versus not only healthy donors, but also versus all other less severe COVID-19 groups. On the other hand, although increased resolvin (RvD5) levels were found in moderate to severe groups versus non-affected individuals, it was not possible to identify patients with a mild course of the disease by SPM analysis. In a systematic review and meta-analysis of 17 randomized trials enrolling 1239 patients, Lu et al.^32^ concluded that Omega-3 supplementation compared to no supplementation or placebo had no effect on mortality, but significantly reduced ICU length of stay and duration of mechanical ventilation of the patients. Other studies carried out on patients with sepsis or multiple inflammatory and respiratory problems have shown a reduction of CRP and IL-6 concentrations after n-3 FA supplementation^29^. Diverging from our hypothesis, our data suggested that this beneficial effect was not associated with changes in PGE_2_ concentration.

PGE_2_ is an immunomodulatory eicosanoid generated by COX that crosstalks with cytokines through several mechanisms^33^. PGE_2_ increases arterial dilation and microvascular permeability, increasing the blood flow into inflamed tissue and regulating cytokine expression in immune cells, such as IL-6^9,34^. It has been reported that during influenza-A virus infection, PGE_2_ was upregulated, leading to inhibition of type I interferon production, and suppressing apoptosis through EP_2_ and EP_4_ receptors, causing an increase in virus replication^33,35^. PGE_2_ activates the Nuclear factor-kappa B (NFκB) in macrophages synergistically with TNFα through EP_2_ receptors, thereby inducing the expression of pro-inflammatory genes that codify COX-2 and Monocyte chemoattractant protein 1 (MCP-1)^33,36^. It has been proposed that lowering PGE_2_ concentrations by inhibiting Microsomal prostaglandin E synthase-1 (mPGES-1) could enhance the host immune response against SARS-CoV-2^37^. Our clinical trial did not identify any difference in PGE_2_ concentrations among groups with varying disease severity. Thus, it is possible that a higher EPA and DHA concentration is necessary to change plasma PGE_2_ concentrations. Moreover, PGE_2_ in plasma is rapidly metabolized and excreted in the urine as 11-α-hydroxy-9,15-dioxo-2,3,4,5-tetranor-prostane-1,20-dioic acid. Consequently, urine PGE_2_ may better reflect its synthesis from AA than PGE_2_ in plasma^38,39^.

A reduction of an important marker of non-enzymatic lipid peroxidation, F_2_-IsoPs (Fig. 1D), was observed in Group 1 compared with Group 4. In another study, urinary 15-F_2r_-IsoPs was found lower in older subjects hospitalized for COVID-19 who were receiving daily IV infusions containing about 6 g EPA + DHA/day for 5 days, leading to the conclusion that n3-FA treatment promoted the reduction of oxidative stress in COVID-19^7^. The high amount of F_2_-IsoPs observed in Group 4 compared with Group 1 can also be due to the enhanced secretion of reactive oxygen species (ROS) by the immune cells^30^, since n-3 FA-derived specialized pro-resolving lipid mediators (SPMs) can blunt ROS production from neutrophils^29^.

EPA, DHA, and their oxylipins formed by enzymatic and non-enzymatic oxidative reactions can exert an anti-inflammatory effect through other mechanisms not thoroughly investigated in our study^29^, given that our focus was on PGE_2_. Actually, the fact that n-3 FA derived SPMs and other oxylipins were not detected in our analysis, does not discard the “resolution hypothesis”^6^, based on the association between SPMs derived from n-3 FA and the severity of COVID-19^40^, considering that SPMs blunt polymorphonuclear cells infiltration^41^ and have an essential role in efferocytosis improvement, reducing the massive infiltration of necrotic cell debris observed *post-mortem* in the lungs of deceased COVID-19 patients^42^. In addition, viral-induced cell debris causes endoplasmic reticulum stress, intensifying the inflammatory cycle^43^ and emphasizing the role of efferocytosis by macrophages in reducing the cytokine storm. Moreover, deficient concentrations of SPMs have been identified in the setting of common human inflammatory lung diseases^25^. The efficacy of dexamethasone in COVID-19 could be partly due to its ability to induce pro-resolving lipid mediators^44^.

As summarized in Fig. 3, EPA may act as a peroxisome proliferator-activated receptor-γ (PPARγ) agonist, inhibiting NFκB translocation to the nucleus, leading to a lower expression of genes that codify the pro-inflammatory cytokines, such as IL-1β and IL-6. It has been reported that the inhibition of NFκB reduces inflammation and increases the survival of mice infected with Sars-CoV^45^. Thus, if n-3 FA can inhibit NFκB translocation, as postulated by other study^23^ it can be suggested that the lower concentration of CRP and IL-6 is a result of NFκB inhibition promoted by n-3 FA (Fig. 3).

Clinical and routine laboratory data at hospital admission were applied to build an algorithm that could predict non-worsening patients during the first two weeks, showing a success rate higher than 99%^5^. However, a multivariate analysis applied to our data showed that no standard of biomarkers could be identified to predict the severity of the disease (Fig. 2), achieving a success rate of classification of only 43%, suggesting the complexity of the SARS-CoV-2 infection in terms of factors associated with the disease prognostic in the conditions evaluated in our study.

The immune response demands the inflammatory condition to control the viral infection. That is, inflammation is an essential tool of the immune cells^46^. EPA and DHA do not primarily act as immune suppressors^23^. Instead, EPA and DHA selectively stimulate the pro-resolving cytokines. Omega-3 fatty acids and anti-inflammatory drugs can show different results according to the moment of their intake. While most studies have supplemented patients during treatment in the hospital, in our study, this protection was present at the time of infection, since blood samples were collected at the hospital admission and no intervention with n-3 FA was made during hospitalization. This aspect can be essential in infection control and must be further investigated to achieve a better protection against future infections.

Finally, our study has some limitations. Other factors, such as the initial charge of viral infection, could have contributed to the severity of the disease. Due to the low amount of sample and their rapid metabolism in plasma, some oxylipins were below the detection limit. As all patients were hospitalized, no comparison was made with patients who were not treated in the hospitals. In addition, we had not information about the use of NSAIDs before the hospitalization. It is worth noting that a higher EPA/AA can also be associated with other factors, including a more diverse and healthy diet^47^, indirectly contributing to a better prognosis of patients during hospitalization. For these reasons, extensive research is needed to confirm our results.

## Conclusion

A higher EPA/AA ratio prior to infection was found to be associated with lower concentration of C-Reactive Protein and interleukin-6, leading to a better prognosis of hospitalized SARS-CoV-2 patients. However, the physiological mechanism of this effect must be further investigated, since a higher EPA/AA ratio was not associated to a lower concentration of oxylipins derived from enzymatic oxidation, as PGE_2,_ but rather to a lower concentration F_2_-IsoP formed through non-enzymatic oxidation.

### Supplementary Information

Supplementary Information.

## Data Availability

Data described in the manuscript, code book, and analytic code will be made available upon request. Requests for data of the study can be sent to Profa. Inar Castro Erger, Department of Food and Experimental Nutrition, Faculty of Pharmaceutical Sciences, University of São Paulo, São Paulo-SP, Brazil (email: inar @usp.br).
