# Supplementary material for: The severity of COVID-19 upon hospital admission is associated with plasma omega-3 fatty acids
Source: Sci Rep. 2024 May 3;14:10238. doi: 10.1038/s41598-024-60815-y (PMC11068876; doi:10.1038/s41598-024-60815-y)
Supplement: Supplementary file 1 — Supplementary Information. [file 41598_2024_60815_MOESM1_ESM.docx]

**
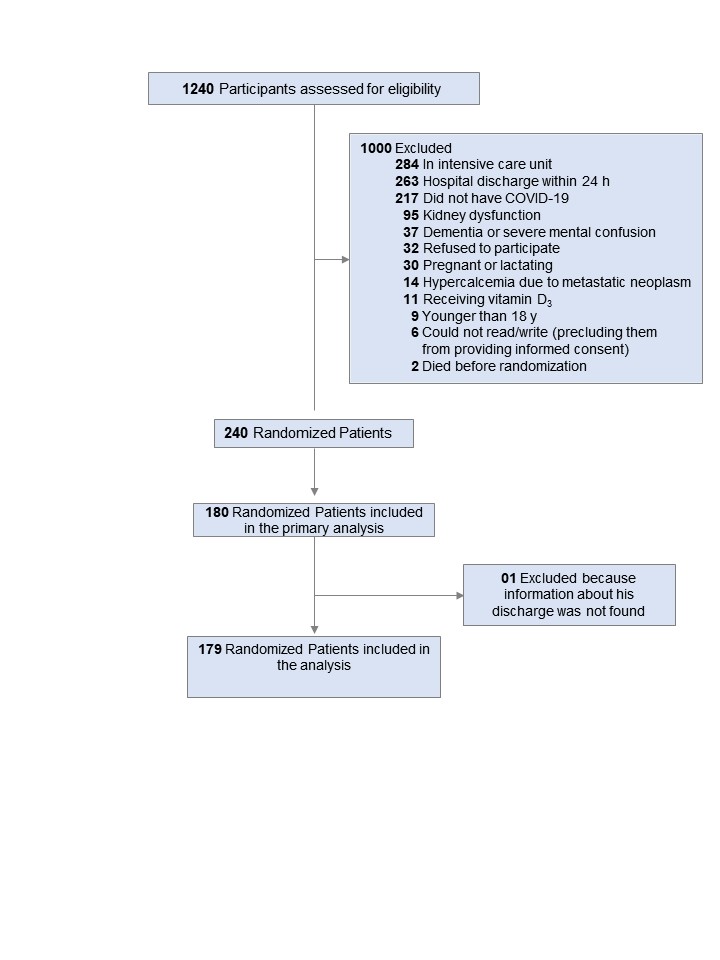
**

**Supplementary Figure 1**. Flowchart

**Supplementary Table 1.** Drugs prescribed to less than 5% of the patients during the hospital stay.

| **Pharmacological treatment during the hospital stay** | **% of the patients** |
| --- | --- |
| Acetylcysteine (200 mg) | 1.67 |
| Alenia (Budesonide,Formoterol) (12/400 µg) | 0.56 |
| Amiodarone (200 mg) | 0.56 |
| Atorvastatin (20 mg) | 1.67 |
| Bactrim (Trimethoprim and Sulfamethoxazole) (400/80 mg) | 0.56 |
| Cabergoline (0.25 mg) | 0.56 |
| Calcium gluconate 10% (10 mL) | 0.56 |
| Carvedilol (3.125 mg) | 3.33 |
| Ceftriaxone (1 g) | 1.11 |
| Chlorthalidone (50 mg) | 1.11 |
| Ciprofibrate (100 mg) | 0.56 |
| Clarithromycin (500 mg) | 0.56 |
| Clonidine (0.1 mg) | 0.56 |
| cloperastine fendizoate (3.54 mg) | 1.67 |
| Clopidogrel (75 mg) | 0.56 |
| Colistin (150 mg) | 0.56 |
| [Cyclobenzaprine](https://www.google.com/search?q=Cyclobenzaprine+treatments+drugs&sa=X&ved=2ahUKEwiY05Dt5p74AhUIqpUCHRu1D5UQ7xYoAHoECAIQNw) (20 mg) | 0.56 |
| Dexchlorpheniramine (2 mg) | 1.11 |
| Diazepam (5 mg) | 1.67 |
| Digoxin (0.28 mg) | 0.56 |
| Dimethicone (40 mg) | 0.56 |
| Dornase alfa | 0.56 |
| Ezetimibe (10 mg) | 0.56 |
| Fenoterol + Atrovent | 0.56 |
| Finasteride  (5.0 mg) | 1.11 |
| Fluoxetine (20 mg) | 0.56 |
| Fluticasone Propionate nasal spray (250 µg) | 1.11 |
| Formoterol fumarate (12 µg) + budesonide (400 µg) | 0.56 |
| Furosemide (40 mg) | 1.67 |
| Glifage  (500 mg) | 0.56 |
| Heparin (5000 UI) | 0.56 |
| Hydralazine (25 mg) | 1.11 |
| Hydrocortisone (100 mg) | 1.11 |
| Hydroxyzine  (10 mg) | 0.56 |
| Ketoprofen (100 mg) | 1.67 |
| Levofloxacin (500 mg) | 0.56 |
| Lidocaine (20 mg/mL) | 0.56 |
| Lithiumcarbonate (300 mg) | 0.56 |
| Loratadine (10 mg) | 0.56 |
| Meropenem  (1 mg) | 0.56 |
| Methylprednisolone (70 mg) | 1,67 |
| Methylprednisolone (80 mg) | 0.56 |
| Metronidazole  (500 mg) | 0.56 |
| Morphine | 0.56 |
| Oseltamivir  (75 mg) | 1.67 |
| Oxacillin (2 mg) | 0.56 |
| Pantoprazole (40 mg) | 0.56 |
| Piperacillin | 0.56 |
| Piperacillin (4 g) and Tazobactam (0.5 g) | 0.56 |
| Prednisone (20 mg) | 1.67 |
| Propranolol (40 mg) | 1.11 |
| Pyridoxine | 1.67 |
| Scopolamine (20 mg) | 1.11 |
| Sertraline  (50 mg) | 2.22 |
| Spironolactone (25 mg) | 1.67 |
| Tacrolimus (1 mg) | 0.56 |
| Tamsulosin (0.4 mg) | 0.56 |
| Tazobactam | 0.56 |
| Terbutaline (500 µg) | 0.56 |
| Thiamine (100 mg) | 0.56 |
| Tiotropium (2.5 mg) | 0.56 |
| Tramadol ( 50 mg) | 2.78 |
| Warfarin | 0.56 |

**Supplementary Table 2**. Analysis of variance of the severity response according to the EPA/DHA ratio factor, adjusted for sex, age and duration of symptoms (time), using the gamma distribution inflated to zero, as proposed by Rigby and Stasinopoulos [49].

Mu link function: log

Mu Coefficients:

Estimate Std. Error t value Pr(>|t|)

(Intercept) -4.224e+00 6.341e+03 -0.001 0.999

Group 1 1.280e+00 6.341e+03 0.000 1.000

Group 2 1.295e+00 6.341e+03 0.000 1.000

Group 3 1.328e+00 6.341e+03 0.000 1.000

Group 4 9.343e-01 6.341e+03 0.000 1.000

Sex -3.921e-03 1.191e-01 -0.033 0.974

Age 4.655e-04 3.775e-03 0.123 0.902

Time 2.713e-02 1.728e-02 1.571 0.118

Removing variables Sex and Age:

Estimate Std. Error t value Pr(>|t|)

(Intercept) -4.28185 9686.34289 0.000 0.9996

Group 1 1.25346 9686.34289 0.000 0.9999

Group 2 1.28756 9686.34289 0.000 0.9999

Group 3 1.33443 9686.34289 0.000 0.9999

Group 4 0.97862 9686.34290 0.000 0.9999

Time 0.03705 0.01641 2.257 0.0253 *


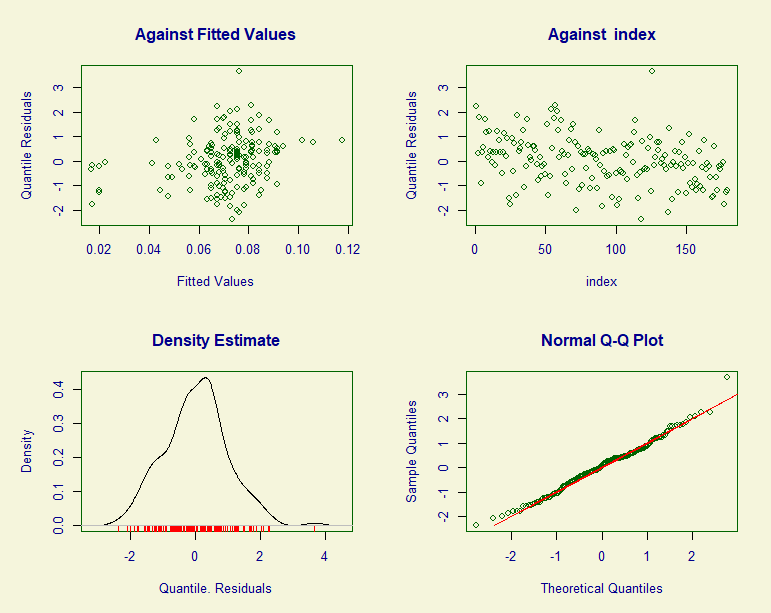


**Supplementary Table 3**. Eigenvectors (n=159 patients x 22 variables)

| Variable | PC*1 | PC2 | PC3 | PC4 | PC5 | PC6 | PC7 | PC8 | PC9 | PC10 |
| --- | --- | --- | --- | --- | --- | --- | --- | --- | --- | --- |
| RBC | 0.125 | -0.530 | 0.177 | 0.049 | 0.303 | 0.126 | 0.018 | 0.065 | 0.221 | -0.096 |
| Hemoglobin | 0.166 | -0.518 | 0.153 | 0.100 | 0.333 | 0.063 | -0.014 | 0.010 | 0.146 | -0.027 |
| Neutrophils | 0.039 | 0.208 | -0.162 | -0.001 | 0.222 | 0.380 | -0.265 | 0.288 | 0.109 | 0.207 |
| Lymphocyte | 0.148 | 0.038 | 0.178 | 0.012 | -0.286 | 0.100 | 0.198 | -0.073 | -0.169 | -0.625 |
| C-reactive protein | -0.252 | -0.110 | -0.041 | -0.219 | -0.031 | 0.256 | 0.029 | -0.065 | -0.254 | 0.080 |
| D-dimer | 0.013 | -0.071 | -0.075 | 0.045 | -0.093 | 0.158 | -0.654 | -0.152 | -0.105 | 0.130 |
| Triacylglycerol | 0.134 | 0.256 | 0.207 | 0.311 | 0.294 | -0.084 | -0.087 | 0.083 | -0.127 | -0.138 |
| IL-6 | -0.039 | -0.024 | -0.094 | -0.416 | 0.334 | 0.022 | -0.015 | 0.084 | -0.390 | -0.063 |
| MDA | -0.009 | -0.094 | -0.302 | -0.105 | 0.358 | -0.157 | 0.111 | -0.065 | -0.354 | -0.230 |
| PGE_2_ | 0.118 | -0.110 | -0.086 | 0.015 | -0.080 | -0.516 | -0.058 | 0.534 | 0.042 | 0.109 |
| 15-HETE | -0.168 | 0.233 | -0.068 | 0.073 | 0.167 | 0.450 | 0.287 | 0.085 | 0.290 | -0.037 |
| 12,13-DiHOME | 0.261 | -0.083 | -0.363 | 0.302 | 0.019 | 0.028 | 0.261 | -0.222 | -0.156 | 0.189 |
| 9,10-DiHOME | 0.289 | -0.062 | -0.342 | 0.266 | -0.100 | 0.064 | 0.278 | -0.079 | -0.076 | 0.290 |
| 5-series-F_2_-IsoP | -0.280 | 0.188 | -0.083 | 0.089 | 0.153 | -0.202 | 0.231 | 0.268 | 0.219 | -0.045 |
| C12:0 | 0.292 | -0.098 | -0.104 | -0.266 | -0.338 | 0.080 | -0.005 | 0.344 | -0.100 | 0.048 |
| C16:1 n7 | 0.355 | 0.231 | 0.018 | 0.068 | 0.150 | 0.056 | -0.129 | 0.255 | -0.058 | -0.160 |
| C17:0 | 0.203 | -0.096 | -0.249 | -0.229 | -0.139 | 0.327 | 0.092 | 0.265 | 0.164 | -0.238 |
| C18:3 n6 | 0.261 | 0.162 | 0.022 | 0.323 | 0.019 | 0.089 | -0.208 | -0.023 | -0.126 | -0.215 |
| C20:5 n3 | 0.372 | 0.203 | 0.160 | -0.339 | 0.130 | -0.064 | 0.098 | -0.213 | 0.148 | 0.148 |
| Age | -0.025 | 0.116 | -0.475 | -0.101 | 0.240 | -0.143 | -0.146 | -0.118 | 0.099 | -0.206 |
| BMI (kg/m^2^) | -0.021 | 0.060 | 0.334 | 0.067 | 0.121 | 0.168 | 0.228 | 0.268 | -0.489 | 0.308 |
| EPA/AA ratio | 0.344 | 0.210 | 0.181 | -0.345 | 0.127 | -0.108 | 0.081 | -0.239 | 0.171 | 0.180 |

*Principal Component

Discriminant Analysis: SEVERITY versus PC1- PC10, using Cross validation

|  | **True classification** | | | | |
| --- | --- | --- | --- | --- | --- |
| **Groups** | **1** | **2** | **3** | **4** | **5** |
| **1** | 29 | 10 | 6 | 1 | 0 |
| **2** | 13 | 22 | 12 | 0 | 1 |
| **3** | 5 | 10 | 13 | 1 | 1 |
| **4** | 4 | 2 | 9 | 3 | 2 |
| **5** | 0 | 4 | 9 | 1 | 1 |
| **Total** | 51 | 48 | 49 | 6 | 5 |
| **Success** | 29 | 22 | 13 | 3 | 1 |
| **Success rate (%)** | 0.569 | 0.458 | 0.265 | 0.500 | 0.200 |

General success rate: 42.80%
